# Diagnosis, risk factors and management of diabetes mellitus in HIV-infected persons in France: A real-life setting study

**DOI:** 10.1371/journal.pone.0250676

**Published:** 2021-05-14

**Authors:** Isabelle Kousignian, Aurélie Sautereau, Corinne Vigouroux, Agnès Cros, Sandrine Kretz, Jean Paul Viard, Laurence Slama

**Affiliations:** 1 Unité de Recherche « Biostatistique, Traitement et Modélisation des données biologiques » BioSTM—UR 7537, Université de Paris, Paris, France; 2 Department of infectious diseases, Assistance Publique-Hôpitaux de Paris, Hôtel Dieu Hospital, Paris, France; 3 Department of infectious diseases, Assistance Publique-Hôpitaux de Paris, Bichat Hospital, Paris, France; 4 Sorbonne Université, Inserm UMR S 938, Saint-Antoine Research Center, University Hospital Institute of Cardio-metabolism and Nutrition (ICAN), Paris, France; 5 Departments of Endocrinology and of Molecular Genetics, Assistance Publique-Hôpitaux de Paris, Saint-Antoine Hospital, National Reference Centre of Rare Diseases of Insulin Secretion and of Insulin Sensitivity (PRISIS), Paris, France; 6 COREVIH Ile de France Sud, Hôpital Henri Mondor, Creteil, France; 7 Centre de Diagnostic et de Thérapeutique, Hôpital Hôtel Dieu, Paris, France; 8 CNRS 8104/INSERM U1016, Institut Cochin, Université de Paris, Paris, France; Centre de Recherche en Cancerologie de Lyon, FRANCE

## Abstract

**Background:**

Diabetes mellitus (DM) is a major and increasing public health problem that may be underdiagnosed and undertreated among persons living with HIV (PLWH).

**Objective:**

To describe the diagnosis, treatment and follow-up of DM among PLWH.

**Methods:**

This study was performed inside a monocentric cohort of 1494 PLWH. DM was defined as having a FG ≥126 mg/dL twice or a HbA1c ≥6.5%, or a history of diabetes, or receiving antidiabetic treatment. The first visit mentioning a diagnosis of DM was considered as the baseline visit. Chi-Square or Fisher exact test were used to examine the association between categorical variables and DM, Wilcoxon or Student t-test were used for continuous variables.

**Results:**

156 PLWH with DM were included. Compared to non-diabetic participants, they were more likely to be native of Sub Saharan Africa (31.6% vs. 22.4%, p = 0.027) and older (54.6 vs. 49.9 years, p<0.001), to have a higher BMI (> 25 for 46.1% vs. 35.3%, p = 0.020) and a poorer control of HIV (HIV RNA<50 copies/mL: 80.1% vs. 89.5%, p<0.001). The diagnosis of DM was missed in 37.8% of PLWH, and 47.2% of PLWH treated for DM did not reach a HbA1c<7%. PLWH with DM were more frequently on antihypertensive and/or lipid-lowering medications: 94.2% had a LDL-cholesterol <70 mg/dL and 60.9% had a blood pressure <140/90 mmHg.

**Conclusion:**

In a setting of HIV-control, HIV care providers should focus on metabolic issues. The management of DM and associated risk factors is mandatory to prevent cardiovascular disease in PLWH.

## Introduction

Diabetes mellitus (DM) is a major public health problem with a worldwide prevalence of 8.5% according to WHO’s (World Health Organization) last report that is expected to increase in the next decades [1]. In 2015, more than 5% of the French population was on glucose-lowering treatment and 25% of persons with diabetes were untreated or ignored their status [2]. People living with HIV (PLWH) are highly impacted by DM, with an estimated prevalence up to 15% [3] and an incidence of 10/1000 person-years, which contributes to an increased risk of cardiovascular diseases [4–6].

Given the improved effectiveness of antiretroviral therapies (ARTs) resulting in a drastic drop in HIV- related conditions, a higher life expectancy but also a longer ART exposure, management of DM is crucial to improve quality of life and avoid complications in PLWH. A previous study has shown that DM is four-fold more common in ART-treated PLWH than HIV-negative persons [7]. Similar to the general population, DM in PLWH has been associated with age, gender, body mass index (BMI), waist circumference, metabolic syndrome, family history of DM, ethnicity as well as dyslipidemia and high blood pressure [3,6,8]. However, specific HIV-related risk factors may contribute to the increased prevalence of DM in PLWH, including cumulative ART exposure [9], use of ‘metabolically unfriendly’ antiretroviral drugs such as first-generation protease inhibitors and nucleoside analogues [10–12], lipodystrophy, as well as prolonged HIV exposure duration, low CD4 nadir [7] and persistent inflammation [3,6,8].

International guidelines for DM management in PLWH are similar to those in the general population. However, the response to glucose-lowering therapies may be poorer in PLWH as compared to the general population [13–15].

The purpose of our study was to identify, in a ‘real life’ setting, the risk factors, circumstances of diagnosis and management of DM in a cohort of PLWH routinely monitored in an academic hospital in Paris, in an attempt to improve the routine management of diabetes in this population.

## Materials and methods

### Study population, inclusion criteria and laboratory analyses

This study was conducted inside the prospective OVIHD cohort [16] (Observatoire VIH de l’Hôtel-Dieu), a single center cohort of 1494 PLWH enrolled since February 11^th^, 2010 at the Hôtel-Dieu University Hospital, APHP, Paris, France. The aim of the OVIHD study is to evaluate the efficacy and safety of combined ART and the occurrence of comorbidities. Participants attend annual visits that include physical examination, longitudinal data regarding all medications received (including antidiabetic and antiretroviral therapies) and blood collection for routine laboratory testing (immunovirological profile, viral hepatitis B and C coinfection status, fasting glucose, HbA1c, fasting lipids and kidney function) and storage. All HIV-infected participants were included if they were adults (age> 18 years old) and gave informed written consent for the use of their socio-demographic, clinical and standard-of-care biological data as well as one of their blood samples to be frozen and stored for further analysis to be used. All data were collected in a centralized database (using NADIS^®^ as computerized medical record) [17]. The OVHID study protocol was approved by the Ethics Committee « Ile de France II » (N° ID RCB: 2010-A00417-32) and by the French general data protection regulation (CNIL). Approval was obtained from our institutional review board and the study was conducted according to the Declaration of Helsinki.

We studied HIV-infected participants, enrolled from February 11^th^ 2010 to December 31^st^ 2018, with a diagnosis of DM, according to the American Diabetes Association (ADA) criteria [18] (defined as having a 8-hour fasting glucose (FG) ≥ 126 mg/dL twice or a HbA1c ≥ 6.5%), or currently being on antidiabetic medication or having a personal history of DM as recorded in the OVIHD NADIS^®^ database. Patients diagnosed with type 1 DM or transient DM were excluded from our analysis. The first visit mentioning a diagnosis of DM was considered as the baseline visit. If DM was diagnosed prior to the inclusion in OVIHD, we used the date of February 11^th^ 2010 as the baseline visit.

Laboratory analyses were performed using standard procedures. Plasma FG levels were measured after at least 8-hour fasting. HbA1c was measured by immunoassay (Cobas, Roche Diagnostics) centralized at the biology laboratory of the Cochin-Hôtel-Dieu hospital, Paris.

### Cardio-metabolic risk factors

DM properly controlled was defined as a HbA1c below 7% at 6 months after DM treatment initiation [18,19]. Patients were defined as having hypertension if receiving antihypertensive treatment, as reported in the NADIS^®^ database or with a confirmed blood pressure ≥ 140/90 mmHg at a subsequent visit. Controlled hypertension was defined a blood pressure ≤ 140/90 mmHg [20,21].

Hypercholesterolemia was defined as being on a lipid-lowering medication as collected in the NADIS database or having low-density lipoprotein cholesterol (LDL-c) > 0.7 g/L. This threshold was chosen since this target was required for most patients with DM at the time of the study.

BMI (weight in kilograms divided by the square of height in meters) was used to define: normal weight (BMI ≤ 25), overweight (BMI between 25 and 30) and obesity (BMI > 30).

Cardiovascular history was recorded as a risk factor in case of personal history of stroke or heart attack.

Renal dysfunction was defined as an estimated glomerular function rate (eGFR) ≤ 60 ml/min using the MDRD equation.

Smoking was considered as a risk factor if patients were current smokers or if they had stopped within the past 3 years.

### Statistical analysis

All statistical analyses were done using RStudio software version 3.5.1 (www.r-project.org). Continuous variables were summarized as the median and interquartile range (IQR), and categorical variables as the frequency and percentage. The Chi-Square test or Fisher exact test was used to determine if there is a significant relationship between categorical variables and the group of patients, and either a Student t-test or a Wilcoxon test to compare the level of continuous variables between the two groups of patients. Statistical tests were two-tailed and p values less than 0.05 were considered to denote statistical significance.

## Results

Between February 11^th^ 2010 and 31^st^ December 2018, a total of 1494 PLWH were included in the OVIHD cohort. They were mostly men (n = 1137, 76.1%), Caucasians (n = 953, 63.8%), with a median age of 50.4 [IQR: 44.9–56.3], a normal BMI for 63.6% of them (n = 947) and a median follow up of 4.6 years [IQR: 2.3–6.6]. At baseline, 98.4% of the population was on ARTs.

**Fig 1:**
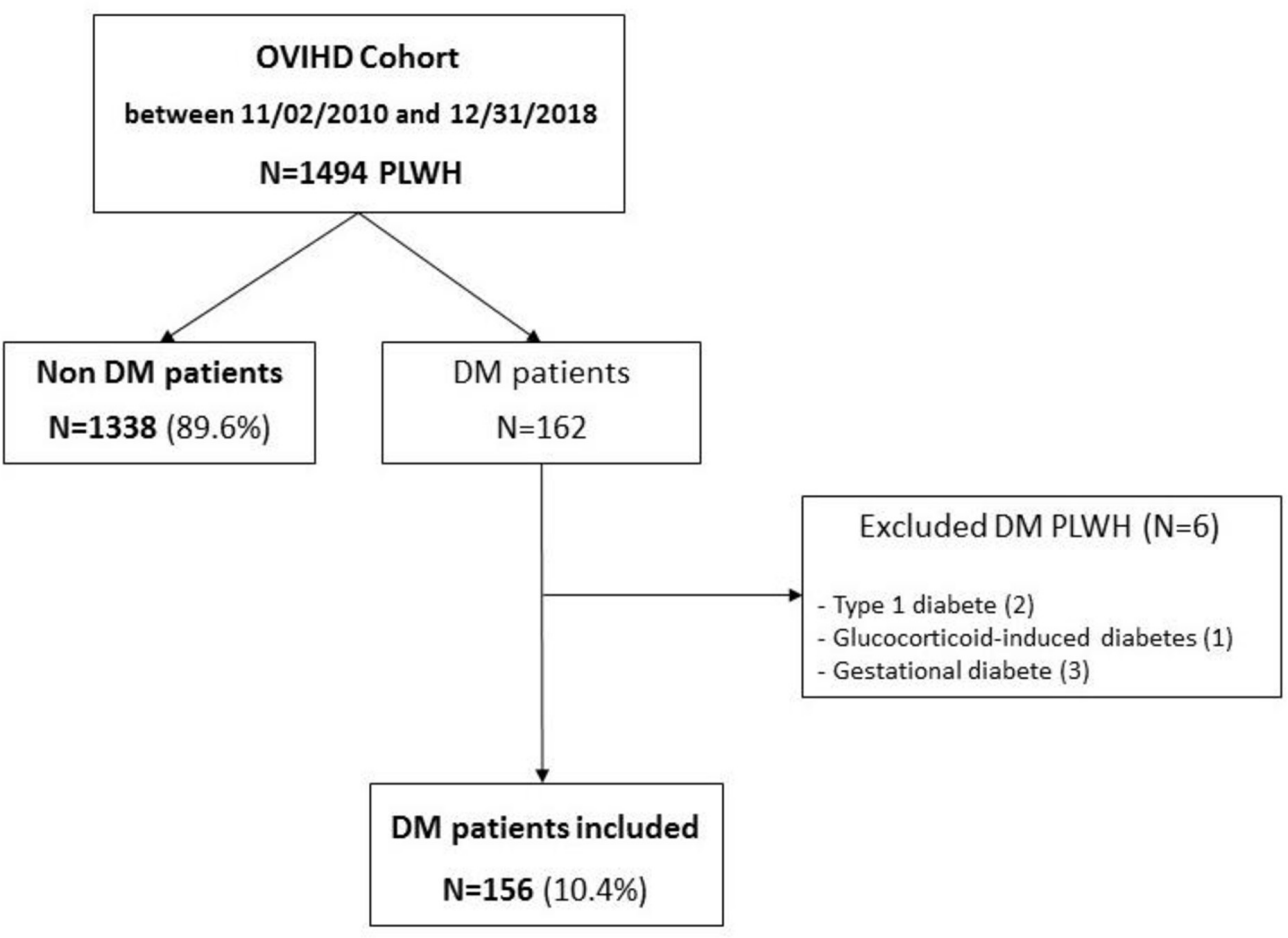
Flow chart of the study population. OVIHD: Observatoire VIH de l’Hôtel-Dieu; PLWH: People living with HIV; DM: Diabetes mellitus.

162 out of 1494 participants were diagnosed with DM. Six patients were excluded from our analysis (two patients with type 1 DM, and four patients with transient DM due to corticosteroid treatment or gestational diabetes). A total of 156 DM patients (10.4% of the entire OVIHD cohort) and 1338 non DM patients (89.6%) were included in our analysis **(Fig 1)**. The median duration of follow up was 5.6 years [IQR: 3.1–7.5] for participants with DM and 4.5 years [IQR: 2.3–6.5] for participants without DM (p<0.001). The median duration of ART exposure was similar in the two groups.

### Characteristics of PLWH at baseline (Table 1)

Similarly to the non DM participants from the OVIHD cohort, DM participants were mostly male (75%). Patients from the DM group were more likely to be native of Sub-Saharan Africa as compared with patients from the non DM group (31.6% and 22.4%, respectively, p = 0.027) and significantly older (median age of 54.6 [IQR: 48.9–62.1] versus 49.9 [IQR: 44.6–55.8], respectively, p<0.001). As expected, DM participants were more frequently overweight or obese (BMI > 25: 46.1% and 35.3%, in the DM and non DM group, respectively, p = 0.020). By crossing data on BMI and region of birth, we found that among patients from Sub-Saharan Africa (23.2% [n = 346] of the OVIHD population), 62.9% (n = 217) were overweight or obese. The route of HIV transmission was different between DM and non DM with 47.4% of heterosexuals in the DM group and 45% of men who have sex with men (MSM) in the non DM group (p = 0.009).

**Table 1:** Characteristics of the study population at baseline.

		OVIHD Cohort N = 1494 patients		
		DM group	Non DM group	P value
		N = 156 (10.4%)	N = 1338 (89.6%)	
Gender (male)		117 (75.0)	1020 (76.3) ^a^	0.771
Age (Years)	Median (IQR)	54.6 (48.9 - 62.1)	49.9 (44.6–55.8)	<0.001
Region of birth	Europe	86 (55.5) ^b^	867 (65.5) ^c^	0.027
	Sub Saharan Africa	49 (31.6)	297 (22.4)	
	others	20 (12.9)	160 (12.1)	
BMI (kg/m^2^)	normal ≤ 25	84 (53.9)	863 (64.7) ^d^	0.020
	overweight >25–30	54 (34.6)	333 (25.0)	
	obesity >30	18 (11.5)	137 (10.3)	
HIV transmission group	heterosexual	74 (47.4)	461 (34.5)	0.009
	MSM	48 (30.8)	603 (45)	
	IDU	12 (7.7)	85 (6.3)	
	Others	16 (10.3)	133 (9.9)	
	Unknown	6 (3.8)	56 (4.2)	
Duration of HIV infection (known)	Median (IQR)	15.2 (9.2 - 22.7)	15.4 (8.6 - 21.9)	0.879
Year of HIV infection	≤1995	72 (46.2)	521 (38.9)	0.188
	1996–2005	52 (33.3)	480 (35.9)	
	≥2006	32 (20.5)	337 (25.2)	
Hepatitis co-infections	hepatitis B	13(8.3)	107 (8.0)	0.876
	hepatitis C	18(11.5))	189 (14.1)	0.446
CDC stage C		36 (23.1)	289 (21.6)	0.748
CD4 cell count nadir (cells/mm^3^)	<200	79 (50.6)	583(43.6)	0.110
Current CD4 cell count (cells/mm^3^)	Median (IQR)	595 (414–851)	590 (423–793)	0.830
	≥500	97 (62.2)	873 (65.2)	0.502
Current viral load (copies/mL)	<50	125 (80.1)	1199 (89.5)	<0.001
	≥50 : Median (IQR)	256 (90–14955)	434 (100 – 13558)	
Patients on ART		149 (95.5)	1321 (98.7)	
Duration of ART exposure	Median (IQR)	12.7 (7.3–17.2)	11.7 (5.8 – 17.0)	0.420

Data are presented as count (proportion on initial sample size) and median (interquartile range).

^a–d^: Values of first and third quartiles; BMI: body mass index, IDU: injecting drug use. MSM: Men who have sex with men; ART: antiretroviral therapy ^a^ with 3 transexual people; ^b–d^:number of missing values; ^b^: 1, ^c^: 14, ^d^: 5.

We found a poorer control of HIV infection in the DM group compared to the non-DM group (HIV < 50 copies/ml in 80.1% and 89.5% of participants, respectively, p< 0.001). No differences were found between DM and non DM participants in terms of cumulative years of known HIV exposure, duration of ART exposure, viral hepatitis co-infections, CDC C-stage or nadir and current CD4 T cell counts.

### The cascade from DM diagnosis to DM control

**Fig 2:**
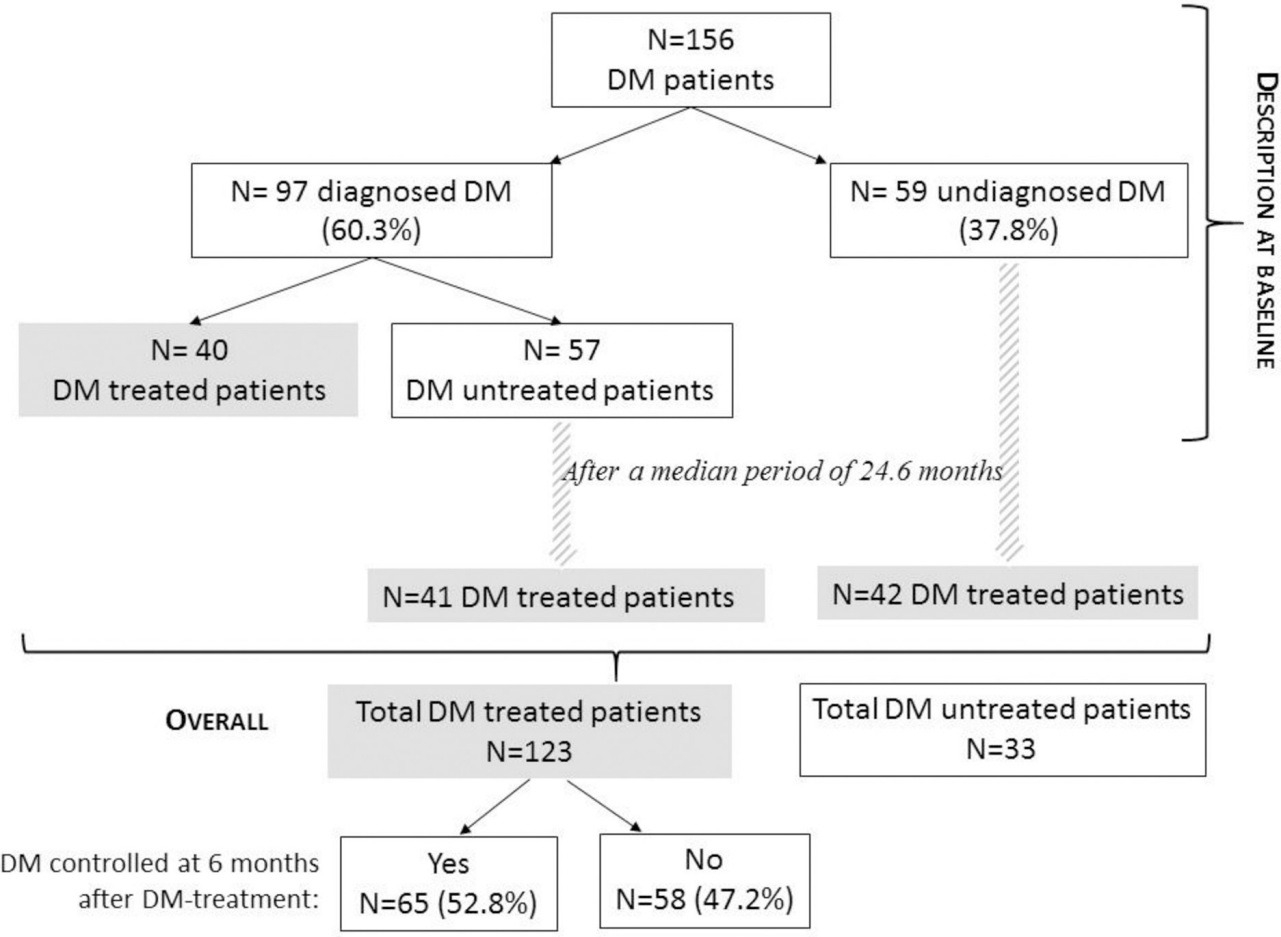
The cascade from diagnosis to DM control. DM treated: Treated by hypoglycemic agents; DM untreated: Without pharmaceutical hypoglycemic treatment.

Among the DM group, 60.3% of patients (n = 97/156) were already diagnosed with DM at baseline: 25.6% of them (n = 40/156) were on glucose-lowering treatment. A HbA1c level below 7% was obtained in 60.9% (25/40) of patients treated with oral antidiabetics **(Fig 2).**

Among patients treated for DM at baseline (n = 40), 65% of participants (n = 26/40) received a monotherapy, mostly metformin, and 22.5% of them (n = 9/40) received sulfonylureas. Few patients received other antidiabetics and/or combined hypoglycemic therapies (1 with metformin and DDP4 inhibitor, 2 with alpha glucosidase inhibitor and 2 with gliptines).

Diabetes was undiagnosed in 59 participants out of 156 (37.8%) at baseline. The DM diagnosis was missed for 29 participants (18.6%) who previously had twice 8-hour FG ≥ 126 mg/dL (≥ 7 mmol/l) and 30 participants (19.2%) who previously had a HbA1c level greater than 6.5%.

Overall, 116 participants were or should have been diagnosed with DM at baseline but remained untreated (57 already diagnosed and 59 participants who should have been diagnosed). From them, within a median of 24.6 months [IQR: 8.7–46.4], 83 participants started a hypoglycemic treatment, mainly based on metformin monotherapy (n = 74, 89.2%). A HbA1c level of less than 7% was reached at 6 months by 52.8% (n = 65/123) of DM participants on hypoglycemic therapy.

### Cardiovascular risk factors and comorbidities at baseline (Table 2)

As expected, DM participants had more cardiovascular risk factors compared to non-DM participants. Although they were less frequently current smokers (21.7% vs. 36.9%, p = 0.001). PLWH with diabetes were more frequently treated with anti-hypertensive (22.4% vs. 14.3%, p = 0.010) and/or lipid-lowering medications (37.8% vs. 24.9%, p< 0.001) and they had more frequently reported personal history of cardiovascular events (5.1% vs.1.9%, p = 0.017). In addition, PLWH with DM presented more frequently renal dysfunction (12.4% and 7.1%, respectively p = 0.030).

**Table 2:** Cardiometabolic comorbidities in the OVIHD cohort.

	DM patients	Non DM patients	P value
	N = 156	N = 1338	
Personal history of Cardio vascular event, n (%)	8 (5.1)	25 (1.9)	0.017
Current smokers, n (%)	33 (21.7) ^a^	477 (36.9) ^b^	<0.001
Renal dysfunction, n (%)	19 (12.4) ^c^	95 (7.1) ^d^	0.030
Anti-hypertensive treatment, n (%)	35 (22.4)	191 (14.3)	0.010
Blood pressure ≤ 140/90 mmHg	95 (60.9)	1178 (88.0)	<0.001
Dyslipidemia treatment, n (%)	59 (37.8)	333 (24.9)	<0.001
LDL-cholesterol < 0.7 g/L	147 (94.2)	1124 (84.3) ^e^	<0.001
LDL-cholesterol < 0.55 g/L	136 (87.2)	739 (55.4) ^e^	<0.001

Data are presented as count (proportion on initial sample size); ^a–e^: number of missing values

^a^: 4

^b^: 48

^c^: 3

^d^: 4

^e^: 6.

Choosing a blood pressure target below 140/90 mmHg to define hypertension control, 60.9% of the DM patients were controlled vs. 88% in the non-DM group (p < 0.001). Setting the target below 130/80 mmHg, hypertension control rates falls to of 32.1% in the DM group (n = 50) and 64.6% (n = 865) in the non-DM group (p< 0.001).

Regarding dyslipidemia, 94.2% of DM participants (n = 147/156) had a LDL-c below 70 mg/dl. Setting the target below 55 mg/dl, only 87.2% (n = 136/156) of the DM participants were controlled. However, diabetics had a better control of dyslipidemia than non DM patients, either with a LDL-c target at 55 mg/dl or at 70 mg/dl (p = 0.001).

## Discussion

Few studies provide a description of diabetes care among PLWH. We show that DM is underdiagnosed and undertreated by hypoglycemic agents in almost 20% in this population, based on the observation of routine care practices, in France. Indeed, in our study, the diagnosis of DM was missed in 37.8% of PLWH, and 47.2% of PLWH treated for DM did not reach the HbA1c goal (< 7%) considered as the optimum objective in most patients.

### Prevalence and risk factors for DM

In developed countries DM prevalence has increased in aging people, including PLWH. Nevertheless, geographical disparities have been well documented [22]. For example, the estimated DM prevalence in the general population reaches 9.4% in the United States vs. 5% in France [18,22,23]. Similarly, DM prevalence in PLWH has been reported to vary from 3 to 9.7% in European cohorts [24–26] whereas other studies reported higher prevalences up to 15% in the United Kingdom, Northern America and Australia [3,5,27,28]. In our study, we found a DM prevalence at 9% (when defined as FG > 126 mg/dL, twice) or 10.4% (when defined as FG > 126 mg/dL twice or HbA1c > 6.5%). These rates are higher than those reported in the Data Collection on Adverse Events of Anti-HIV Drug (D:A:D) study (3%) [29] and the EuroSIDA study (6.3%) [30] but similar to other published data from HIV cohorts in Western Europe [24,25,31].

Age is a well-known risk factor for DM [32]. However, the prevalence of DM at a younger age is likely to be higher in PLWH than in the general population in most countries including France [20]. This is true even when the prevalence of DM in the general population in France includes persons with untreated and/or undiagnosed DM [2,33]. The higher DM prevalence in PLWH may be explained by an over-representation of risk factors such as older age, male gender, higher BMI or minority ethnicity [1,2,32,34,35]. However, in our study, PLWH with DM were older than their non DM counterparts (54.6 vs. 49.9 years old, respectively) but younger than people with DM in the French general population [2,36]. The OVID cohort is mainly composed of men (76.1%). However, in our study, the proportion of men is not significantly different between DM and non-DM PLWH.

In our study, participants with DM had more frequently a BMI above 25 than non DM participants. However, obese persons were underrepresented in our study (21.8%) compared to the French DM population, in whom the prevalence of obesity is around 41% [33]. In a meta-analysis of 44 studies, Nansseu [6] reported a higher burden of DM among patients treated for HIV with an incidence rate of 13.7 per 1,000 person years of follow up. Similar to our findings, aging and overweight were reported as major risk factors for DM [2]. However, we did not find any differences in terms of cumulative years of known HIV exposure or duration of cART exposure previously reported as major contributor to hyperglycemia [7], except for stavudine (47.7% and 36.4% ever exposed to d4T, in the DM group and non DM group respectively, p< 0.01), between DM and non DM PLWH. Indeed, the first generation of antiretroviral drugs have been shown to favor metabolic dysfunction [37], including insulin resistance syndromes, dyslipidemia, lipodystrophy and DM [10]. Considering the multiple confounding factors associated to combined regimen and the length of patient follow-up, these factors were difficult to identify more specifically.

In addition to traditional risk factors, HIV-specific risk factors may contribute to the high prevalence of DM in our cohort of PLWH. We did not find a lower CD4 cell count nadir in the DM group even if it was previously reported as a risk for decreased insulin sensitivity and/or developing DM in PLWH [7,10,38]. However, our study suggests that uncontrolled plasma viral load could contribute to the risk of DM.

The route of HIV transmission seems associated with DM in our study. However, this association could be a result of confusion factors such as BMI. In fact, heterosexuals with DM had a higher BMI (63.9% with BMI > 25) than MSMs with DM (19.4%), p< 0.001.

Finally, it has been recognized that the rate of several non AIDS complications including DM could be different among ethnic groups [39]. In our study, patients with DM were less frequently native of Europe than patients without DM. However we did not study the role of socioeconomic biases that could contribute to these differences.

### DM diagnoses tools in HIV-infected patients

French guidelines define DM according to WHO criteria, i.e. having a 8 hours FG level above 126 mg/dl (7 mmol/l) twice, or a plasma FG above 200 mg/dl (>11.1 mmol/l) at 2-hours of an oral glucose tolerance test (OGTT) or during a random analysis, with evocative clinical signs (polyuria, polydipsia, loss of weight) [19,34].

In the French general population, it has been recognized that around 20% of persons aged 18 to 74 with FG levels meeting the criteria for DM ignore their DM status. However, the proportion of underdiagnosed DM decreases with age (30% between 30 and 54 and 13% between 55 and 74). In our study, 18.6% of DM PLWH with a median age of 54.6 ignored their DM status, similarly to the general population in France [2].

In the last decade, the American Diabetes Association (ADA) introduced the threshold of 6.5% of HbA1c as an additional screening test for the diagnosis of DM [18]. In our study, almost 20% of PLWH with a missed diagnosis of diabetes (n = 30) could have been diagnosed on the basis of their HbA1c greater than 6.5%. Despite the fact that HbA1c may underestimate FG in PLWH [40–42], our results, supported by other studies [43,44], suggest that HbA1c, in addition to FG or a 2-hour OGTT could be useful for the diagnosis of DM in PLWH. Further studies are needed to determine whether a cutoff below 6.5% should be considered in that setting.

French and international guidelines define DM control as reaching a HbA1c value below 7% for most patients. In France, between 2001 and 2007, in the general population of patients treated with antidiabetic drugs (n = 9781), 41% did not reach this goal [33]. Our findings are consistent with these data since in 55 patients out of 123 under antidiabetic drugs (44.7%) did not reach the 7% goal of HbA1c. These figures are higher than those from a previous study conducted by Satlin et al. among 142 PLWH from New York in which 33% of patients on hypoglycemic therapy (IC_95%_ 25%-42%) had a HbA1c > 7.5% [15]. Similarly to our findings, it has been recognized that a significant rate of DM people in France should have a reconsideration of their DM treatment combination therapy to reach the HbA1c goal below 7% [33,45].

Focusing on DM drugs use in our study, it appeared that the treatment regimens were different to those observed in the French general population: 88.6% vs. 43% of patients with DM were on a monotherapy (mostly metformin) in the OVIHD and in the general population, respectively, and only 11.4% of our PLWH with DM were on a combined oral therapy or more recent drugs [33,46]. In a study performed by Han et al. in American veterans, 50% of participants received metformin [14]. Also, some patients were probably controlled on diet alone but we were unable to collect this data.

### Prevalence and management of cardio-metabolic comorbidities among PLWH with DM

DM represents one of the multiple cardiovascular risk factors leading PLWH to have a higher rate of cardiovascular diseases, occurring at an earlier age, than the general population [4,47]. In our study, we found a lower rate of current smokers in DM (21.7%) compared to non DM (36.9%) participants (p< 0.001), similar to the general population (smokers are 18% among French patients with DM [45] and 36.4% in the general population [48]). This probably reflects the awareness about the risks of tobacco smoking among diabetics.

As expected, a higher proportion of PLWH with DM were treated by anti-hypertensive drugs and statins as compared to non DM participants. Antihypertensive drugs were used in 29.4% of the DM patients from the present study, which is higher compared to 14% reported in the DAD study [29] but similar to what was found in Eurosida [30]. However it is lower than the prevalence of 75% in the ENTRED study [45], representative of patients with DM in France. Almost 61% of our participants were controlled for their hypertension (blood pressure < 140/90 mmHg), while among patients with anti-hypertensive treatment, in the French general population, only 55% have a controlled arterial pressure below 140 mmHg [49]. In our study, 37.8% of PLWH with DM were treated by statins compared to 42% in the DAD study [29] and 45% in the Eurosida study [30], 94.2% of DM participants reached a LDL-c level below 70 mg/dl and. Recently, Clement et al showed in a cohort of HIV/HCV co-infected patients that only 22.7% out of 50.7% of patients who required statins reached the LDL-c target value [50].

We found that the prevalence of renal dysfunction was higher in DM participants than non DM participants (12.4% vs. 7.1%, p = 0.030), but lower compared to the general population (19%) [45]. Obviously, the impact of uncontrolled comorbidities has several long term consequences including renal impairment.

Our study has several limitations. Clinical and biological data were already collected in our database as well as DM medication, with a retrospective analysis. Moreover, we were unable to provide the duration of DM. However by analyzing the routine management of cardio-metabolic risk factors in an urban academic HIV clinic, our study points to factors that could rapidly improve the patient care in a real-life setting.

## Conclusion

Despite excellent HIV control, it is necessary to increase awareness of HIV care providers on metabolic issues. In particular, despite a regular monitoring of glucose levels, the diagnosis of DM is missed in almost 20% of patients. We may also consider HbA1c as a tool for DM diagnosis. Management of DM associated risk factors is also mandatory to avoid cardiovascular diseases in PLWH. Furthermore, our data suggest that screening for DM should be carried out at a substantially earlier age for PLWH compared with HIV uninfected patients, possibly a decade in advance and that the therapeutic management of diabetes should be strengthened.

## Supporting information

S1 Data (7Z)
